# Functional and pharmacological characterization of an S5 domain hERG mutation associated with short QT syndrome

**DOI:** 10.1016/j.heliyon.2019.e01429

**Published:** 2019-04-20

**Authors:** Andrew Butler, Yihong Zhang, A. Graham Stuart, Christopher E. Dempsey, Jules C. Hancox

**Affiliations:** a School of Physiology, Pharmacology and Neuroscience, Medical Sciences Building, University Walk, Bristol, BS8 1TD, United Kingdom; b Bristol Heart Institute, University of Bristol, Bristol, BS2 8HW, United Kingdom; c School of Biochemistry, Medical Sciences Building, University Walk, Bristol, BS8 1TD, United Kingdom

**Keywords:** Biophysics, Computational biology, Physiology

## Abstract

Congenital short QT syndrome (SQTS) is a repolarization disorder characterized by abbreviated QT intervals, atrial and ventricular arrhythmias and a risk of sudden death. This study characterized a missense mutation (I560T) in the S5 domain of the hERG K^+^ channel that has been associated with variant 1 of the SQTS. Whole cell patch clamp recordings of wild-type (WT) and I560T hERG current (I_hERG_) were made at 37 °C from hERG expressing HEK 293 cells, and the structural context of the mutation was investigated using a recently reported cryo-EM structure of hERG. Under conventional voltage clamp, the I560T mutation increased I_hERG_ amplitude without altering the voltage-dependence of activation, although it accelerated activation time-course and also slowed deactivation time-course at some voltages. The voltage dependence of I_hERG_ inactivation was positively shifted (by ∼24 mV) and the time-course of inactivation was slowed by the I560T mutation. There was also a modest decrease in K^+^ over Na^+^ ion selectivity with the I560T mutation. Under action potential (AP) voltage clamp, the net charge carried by hERG was significantly increased during ventricular, Purkinje fibre and atrial APs, with maximal I_hERG_ also occurring earlier during the plateau phase of ventricular and Purkinje fibre APs. The I560T mutation exerted only a modest effect on quinidine sensitivity of I_hERG_: the IC_50_ for mutant I_hERG_ was 2.3 fold that for WT I_hERG_ under conventional voltage clamp. Under AP voltage clamp the inhibitory effect of 1 μM quinidine was largely retained for I560T hERG and the timing of peak I560T I_hERG_ was altered towards that of the WT channel. In both the open channel structure and a closed hERG channel model based on the closely-related EAG structure, I560T side-chains were oriented towards membrane lipid and away from adjacent domains of the channel, contrasting with previous predictions based on homology modelling. In summary, the I560T mutation produces multiple effects on hERG channel operation that result in a gain-of-function that is expected to abbreviate ventricular, atrial and Purkinje fibre repolarization. Quinidine is likely to be of value in offsetting the increase in I_hERG_ and altered I_hERG_ timing during ventricular APs in SQTS with this mutation.

## 1 Introduction

Cardiac action potential (AP) repolarization is mediated by a number of distinct potassium (K^+^) ion channels. The initial early repolarization phase involves transient outward potassium current (I_to_), the pore forming subunits of which are encoded by *KCND2*, *KCND3*, and *KCNA4* genes [1, 2]. Over AP plateau voltages the rapid and slow delayed rectifier K^+^ currents (I_Kr_ and I_Ks_) respectively play critical roles and are therefore important determinants of AP duration, with pore-forming subunits encoded by *hERG* (*human-Ether-à-go-go Related Gene*, alternative nomenclature *KCNH2*) and *KCNQ1* [1,3]. Terminal repolarization is driven by inwardly rectifying K^+^ current (I_K1_), which also plays an important role in setting the resting potential in non-pacemaker regions, through channels comprised of Kir2.x channel subunits [1, 2, 4]. In atrial myocytes an additional K^+^ current, the ultrarapid delayed rectifier, I_Kur_, is also important [1]. In the time since hERG was first identified as underlying the channels that mediate I_Kr_ [5, 6], loss-of-function *hERG* mutations have been found to be responsible for the LQT2 form of congenital long QT syndrome (LQTS), whilst the unique pharmacological promiscuity of the channel has been implicated in drug-induced LQTS [7]. Since 2004, gain-of-function *hERG* mutations have been found in congenital short QT syndrome (SQTS) [8, 9].

The congenital SQTS was first identified as a distinct syndrome in 2000 [10]. SQTS is characterised by: short rate corrected QT (QT_c_) intervals on the electrocardiogram; poor rate adaptation of the QT interval; shortened effective refractory periods; atrial and ventricular arrhythmias and, often, by a history of sudden death in affected families [9, 11, 12]. Mutations in a number of different ion channel subunits have been identified in SQTS patients, although a significant proportion of patients with a SQTS phenotype have not shown mutations in known candidate ion channels [9, 11, 12]. The first successfully genotyped SQTS variant (SQT1) was found to involve a gain-of-function mutation to hERG (N588K, located in the S5-Pore linker region) in which voltage-dependent inactivation was greatly positive-shifted, resulting in a substantial increase in I_Kr_ and a shift in its timing to earlier in the ventricular AP [8, 13, 14, 15]. The profound inactivation shift renders N588K-hERG channels and SQT1 patients with this mutation comparatively insensitive to Class III antiarrhythmic drugs such as sotalol, whilst Class Ia drugs including quinidine and disopyramide retain effectiveness [8, 16, 17, 18]. A second hERG mutation (T618I; located in the channel pore helix) produces a less profound shift in hERG current (I_hERG_) inactivation [19, 20] and together with N588K, accounts for the majority of successfully genotyped cases (respectively accounting for 25.9% and 18.5% of genotyped cases; [9]). Several additional hERG mutations have been associated with the syndrome (E50D; I560T; S631A; R1135H [12, 21, 22, 23]). The I560T mutation was identified in a 64 year old male who presented with atrial fibrillation and flutter and whose father and brother had died suddenly [12]. He had a QT_c_ interval of 319 ms (heart rate of 68 beats min^−1^); the I560T mutation, involving a residue located in the S5 transmembrane helix, was due to a c1679T > C transition and was absent in the genomic DNA of 200 controls [12]. Mature (fully glycosylated) I560T hERG channels were found to express at similar levels to the wild-type (WT) channel and limited *in vitro* biophysical characterisation showed an increase in I_hERG_ magnitude associated with a modest (+14 mV) shift in steady-state voltage dependence of inactivation [12]. Effects of the mutation on time dependent properties of I_hERG_ were not reported, nor was the sensitivity of I560T-hERG to antiarrhythmic agents reported. Prior alanine-mutagenesis has suggested hydrophobic, energetic coupling between S5 and S4 helices of hERG during inactivation involving residue I560 [24], whilst a proximate residue (H562) has been suggested to interact with the channel pore and influence the selectivity filter [25]. It is thus possible that effects of the SQT1 I560T mutation are not restricted to the voltage-dependence of I_hERG_ inactivation. The present study was undertaken to provide a detailed characterisation of the effects of the I560T mutation on I_hERG_ kinetics, on the current's profile during physiological (AP) waveforms and on sensitivity of the channel to the antiarrhythmic drug quinidine. In addition, the recent availability of a hERG structure [26], obtained from cryo-electron microscopy (cryo-EM), enabled this study to consider the functional consequences of the I560T mutation in the context of the hERG channel structure.

## 2 Methods

### 2.1 Mutagenesis

The I560T hERG mutation was generated by PCR-based substitution by Mutagenex Inc (Suwanee, GA 30024, USA) from a wild-type (WT) construct in modified pcDNA3. Competent DH5*α Escherichia coli* (Invitrogen, Paisley, UK) were transformed using standard procedures, DNA was purified by Endotoxin-free plasmid DNA purification kit (Neumann-Neander-Str., Germany, Macherey-Nagel), the mutation was confirmed by sequencing of the entire open reading frame (Eurofins MWG Operon, Ebersberg, Germany).

### 2.2 Cell culture and transfection

Human embryonic kidney (HEK 293) cells with no native I_hERG_ (European Collection of Cell Cultures, Porton Down, UK) were used to study the effects of the I560T mutation on I_hERG_ kinetics and the current's response to AP waveforms. These cells were maintained at 37 °C, 5% CO_2_ in Dulbecco's minimum essential medium with Glutamax-1 (DMEM; Gibco, Paisley, UK). This was supplemented with 10% fetal bovine serum, 50 μg ml^−1^ gentamycin (Gibco, Paisley, UK). Cells were transiently transfected with 1μg cDNA plasmids encoding WT or mutant hERG using Lipofectamine 2000 (Invitrogen, Paisley, UK) according to the manufacturer's instructions. Due to the increased macroscopic I_hERG_ seen in cells expressing mutant channels, only 0.15μg cDNA plasmid encoding I560T was added for protocols in which I_hERG_ inactivation was measured: this prevented excessively large currents from forming and facilitated accurate recordings. Expression plasmid encoding CD8 (0.15μg) was also added (in pIRES, donated by Dr I Baró, University of Nantes, France) to be used as a successful marker of transfection [27, 28]. Electrophysiological recording experiments were performed 12–48 h after transfection (a range within that of prior studies from our laboratory [28, 29]). Successfully transfected cells (positive to CD8) were identified using Dynabeads® (Invitrogen, Paisley, UK; e.g. [27, 28, 30]).

**Fig. 1:**
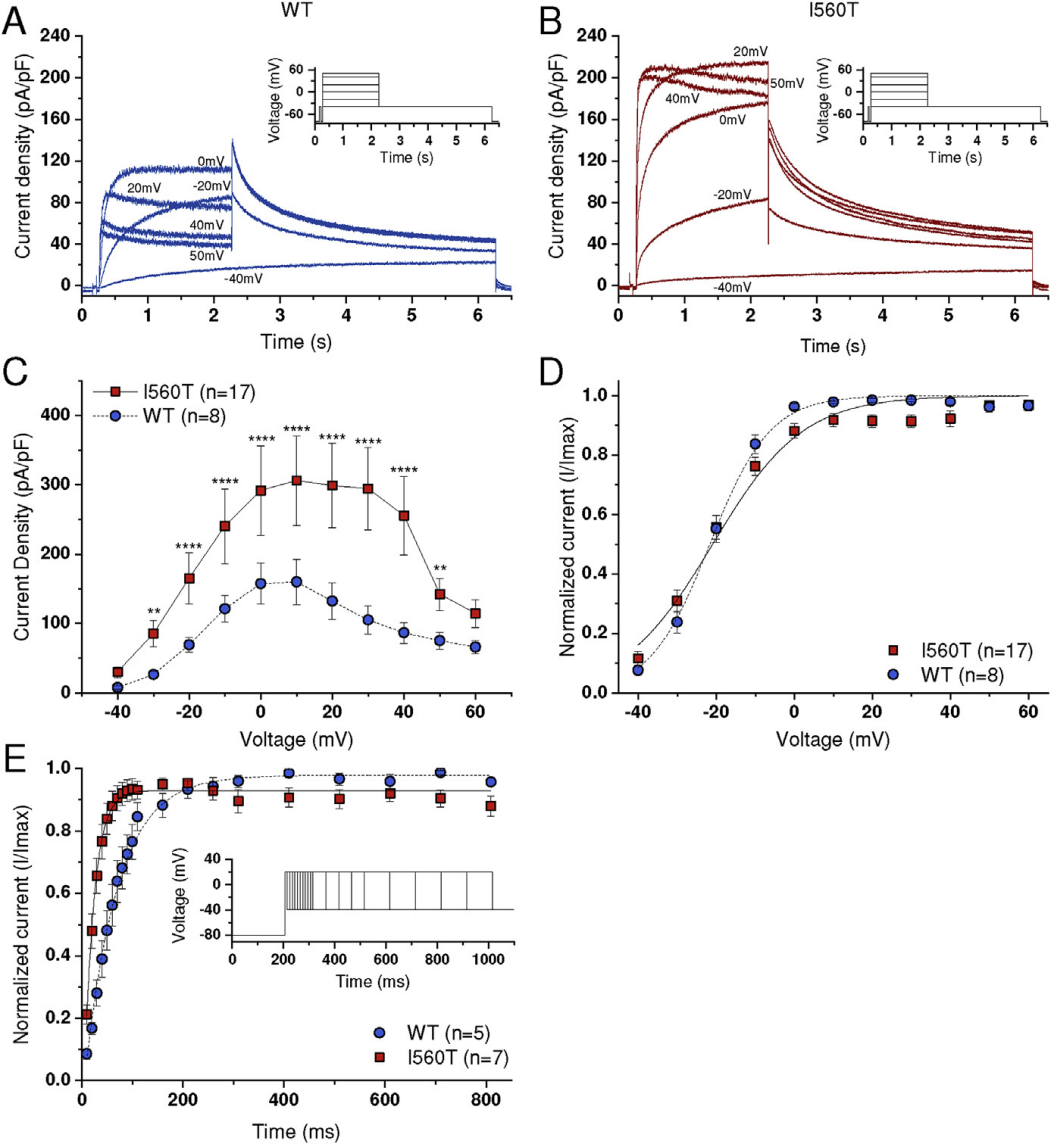
*Current-voltage (I-V) relations for WT and I5*60T hERG *and time-course of I*_*hERG*_*activation.* (A, B) Representative current traces for WT (A; blue) and I560T (B; red) I_hERG_ elicited by the voltage protocol shown as insets. Test pulses were applied at 10 mV increments between −40 mV and +60 mV, with only selected traces being shown for clarity and corresponding test potentials are indicated next to relevant current records. (C) Mean I-V relations for end pulse WT and I560T I_hERG_. ∗∗ denotes significant difference from control at p < 0.01, whilst ∗∗∗∗ denotes significance at p < 0.0001. (D) Mean normalized tail current I-V relations. Currents were normalized to the peak current recorded during the protocol for each cell, and fitted by Eq. 1. (E) Activation time course of WT and I560T I_hERG_ at +20 mV elicited using an envelope of tails protocol (inset), fit with Eq. (2). Replicate numbers are given in each panel.

HEK 293 cells transiently transfected with I560T hERG were also used in the study of the channel's sensitivity to antiarrhythmic drugs. However HEK 293 cells stably expressing WT hERG (generously donated by Dr Craig January, University of Wisconsin, that have been used in multiple prior pharmacological studies from our laboratory (e.g. [31, 32, 33]) were used for comparison of drug block with I560T under conventional voltage clamp (cf [28, 29]), as comparison of absolute ‘control’ current magnitudes was not required between WT and mutant conditions in those experiments, with each cell providing its own control I_hERG_ magnitude prior to drug application (Fig. 6A,B; in contrast with Figs. 1C and 5). These cells were passaged using enzyme free cell dissociation solution (Millipore, Watford, UK) and plated onto sterilized 13-mm glass coverslips in 40-mm Petri dishes containing a modification of Dulbecco's minimum essential medium (DMEM) with Glutamax-1 (Gibco, Paisley, UK). This was supplemented with 10% fetal bovine serum, 50 *μ*g/mL gentamycin (Gibco), and 400 *μ*g/mL geneticin (G418, Gibco). The cells were incubated at 37 °C (5% CO2) for a minimum of 1 day before any electrophysiological study [30].

### 2.3 Solutions for electrophysiological recordings

Once in the recording chamber, cells were superfused with normal Tyrode's containing (in mM): 140 NaCl, 4 KCl, 2.5 CaCl_2_, 1 MgCl_2_, 10 Glucose, and 5 HEPES (titrated to pH of 7.45 with NaOH) [15, 18, 28]. Patch-pipettes were fire-polished to 2.5–4 MΩ. The pipette dialysis solution for hERG current (I_hERG_) measurement contained (in mM): 130 KCl, 1 MgCl2, 5 EGTA, 5 MgATP, and 10 HEPES (titrated to pH of 7.2 with KOH) [15, 18, 28]. Quinidine powder (Quinidine gluconate salt; Sigma-Aldrich, Gillingham, UK) was dissolved in Milli-Q water to produce an initial stock solution of 10 mM, which was serially diluted to produce stock solutions ranging from 10 mM to 1μM.

### 2.4 Experimental protocols and analysis

Measurements of hERG current (I_hERG_) were made at 37 °C ± 1 °C as described previously [15, 18, 28, 33]. Whole-cell patch clamp recordings of membrane currents were made using an Axopatch 200A amplifier (Axon Instruments, Foster City, CA, USA) and a CV201 head stage. Between 70% and 80% of the electrode series resistance could be compensated. Data were recorded via a Digidata 1440A interface (Molecular Devices, Sunnyvale, CA, USA); a bandwidth of 2–10 kHz was set on the recording amplifier and digitization rates up to 25 kHz were used. Data were analyzed using Clampfit 10.2 (Axon Instruments), Excel 2016 (Microsoft, Redmond, WA), Origin 2017 (OriginLab Corporation, Northampton, MA, USA), and Prism 7 (Graphpad Inc, La Jolla, CA, USA) software.

The specific voltage protocols used experimentally are detailed within the relevant figures and associated Results. All action potential (AP) waveforms used here for AP voltage clamp (‘AP clamp’) experiments have been used in prior studies and currents elicited under AP clamp were corrected online for P/N leak subtraction using an interspersed P/4 protocol [28, 30, 34, 35]. Charge carried by WT and mutant hERG channels during AP commands was determined by integrating currents using Origin 2017.

Half-maximal activation (V_0.5_) voltages were obtained by normalizing I_hERG_ tail current values (I), following differing voltage commands, to the maximal I_hERG_ tail value observed during the voltage protocol (I_max_), plotting the resulting values against corresponding command voltage (V_m_), and fitting the data with a Boltzmann equation of the form:

(1) $$\text{I}={\text{I}}_{\text{max}}/\left(1+\text{exp}\left(\left({\text{V}}_{0.5}-{\text{V}}_{\text{m}}\right)/k\right)\right)$$

where *k* is the slope factor describing I_hERG_ activation.

The rate of I_hERG_ activation derived from application of an envelope of tails protocol was obtained by fitting the plotted data with an exponential equation of the form:

(2) $$\text{I}={\text{I}}_{\text{max}}\left(1-\exp\left(-K\;\text{x}\right)\right)$$

Where I is current at time x, where x represents duration of the depolarising step after which tail current amplitude was measured. Values of I were normalized to maximal current I_max_ obtained during the protocol. *K* is the rate constant of activation, from which the time constant was derived as 1/*K*.

Half maximal inactivation voltage (V_0.5_) was obtained from normalized plots of voltage-dependent availability, using the following equation:

(3) $$1-\left(1/\left(1+\exp\left[\left({\text{V}}_{0.5}-{\text{V}}_{\text{m}}\right)/k\right]\right)\right)$$

**Fig. 2:**
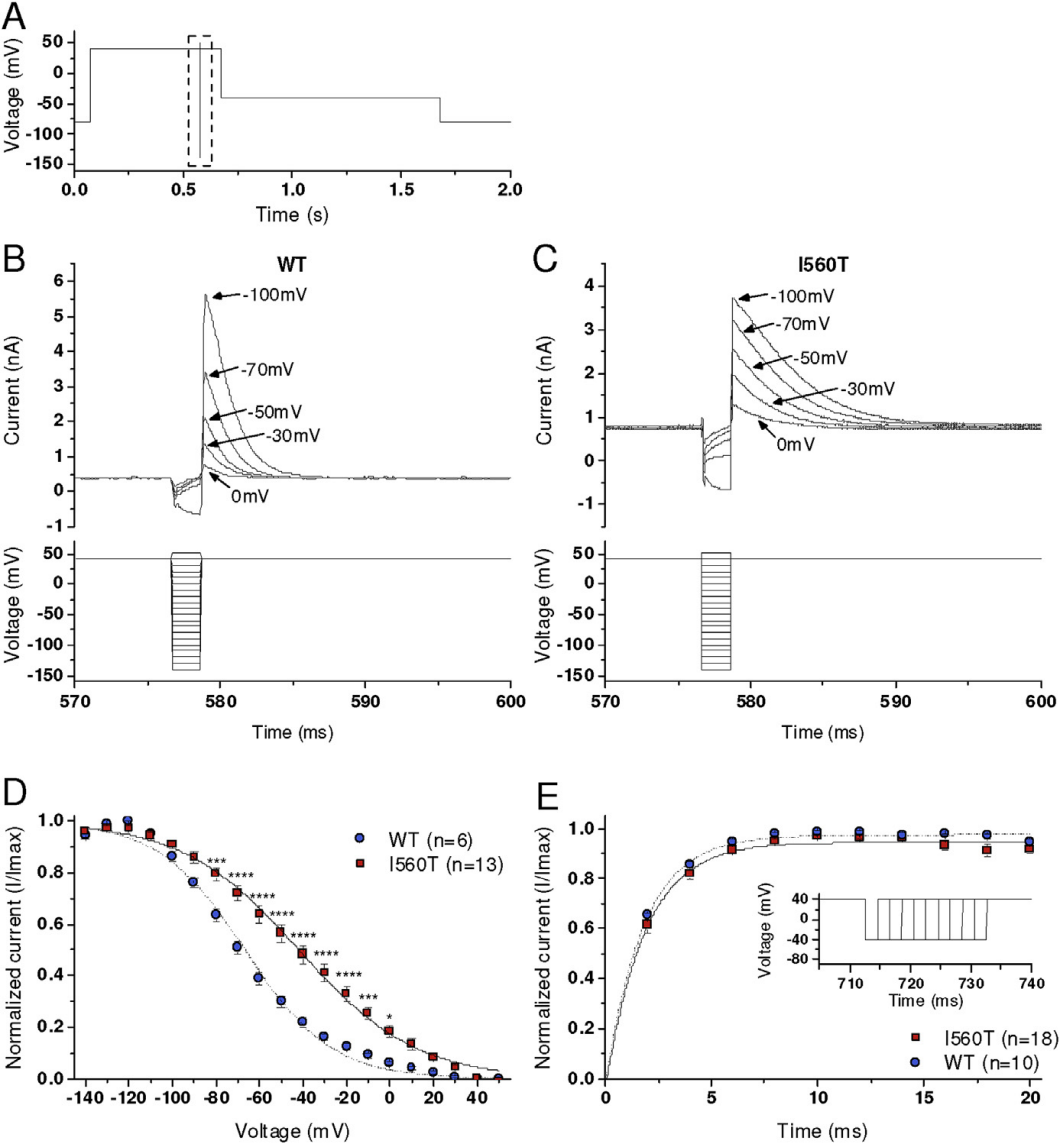
*Inactivation properties of WT and I560T I*_*hERG*_*.* (A) Voltage protocols used to determine I_hERG_ inactivation. The highlighted section is that magnified in lower panels of B and C. (B, C) Representative traces for WT (B) and I560T (C) I_hERG_ aligned with section of protocol shown below. Brief (2 ms) repolarising steps to potentials between -140 mV to +50 mV were used to elicit current, with only selected traces shown here for clarity. Preceding repolarisation step is indicated for each trace. (D) The voltage dependence of inactivation (availability). Currents were normalized to the peak current recorded during the protocol for each cell and fitted with Eq. (3). For currents at the most negative voltages, deactivation correction was performed as described previously [15, 28]. V_0.5_ and *k* values are given in the Results text. Significant difference from control at p < 0.05, p < 0.001 and p < 0.0001 are represented by *, *** and **** respectively. (E) Time-course for the recovery from inactivation for WT and I560T hERG, calculated from 40 mV currents elicited in 2 ms steps up to 20 ms. Recovery time constants are given in the Results text. Replicate numbers are given in each panel.

Where V_m_ represents the repolarization voltage used to influence I_hERG_ availability (Fig. 2, Results) and *k* is the slope factor describing I_hERG_ inactivation.

As previously [28], the I_hERG_ window was obtained from derived activation and inactivation V_0.5_ and *k* values by using equation (3) and a modified version of Eq. (1) to calculate activation/inactivation variables at 2 mV intervals and then the product of the two relations plotted to ascertain the I_hERG_ window.

The deactivation rate of I_hERG_ was quantified by fitting tail currents with the following bi-exponential equation:

(4) $$\text{I}={\text{A}}_{\text{f}}\;\exp\left(-\text{x}/\tau_{\text{f}}\right)+{\text{A}}_{\text{s}}\;\exp\left(-\text{x}/\tau_{\text{s}}\right)+\text{C}$$

where I represents the current amplitude at time x; A_f_ and A_s_ represent the total current fitted by the fast and slow components of the deactivation time-course (τ_f_ and τ_s_ respectively) and C represents any residual unfitted current.

Fractional inhibition of I_hERG_ by quinidine was calculated by:

(5) $$\text{Fractional}\;\text{inhibition}\;=1-\left({\text{I}}_{\text{hERG}}\text{[Drug]}/{\text{I}}_{\text{hERG}}\text{[Control]}\right)$$

where I_hERG_[Drug] and I_hERG_[Control] represent “tail” current amplitudes in the presence and absence of pharmacological agents respectively.

Concentration response relations were fitted by a standard Hill equation of the form:

(6) $$\text{Fractional}\;\text{inhibition}\;=1/\left(1+{\left({\text{IC}}_{50}/\text{[Drug]}\right)}^{h}\right)$$

where IC_50_ is [Drug] producing half-maximal inhibition of the I_hERG_ tail and *h* is the Hill coefficient for the fit.

Unless stated otherwise in the “Results” text, all data are presented as the mean ± standard error of the mean (SEM). Statistical comparisons were made, as appropriate, using a Student's *t*-test (where appropriate with Welch's correction), Mann-Whitney U test or one- or two-way analysis of variance (ANOVA) followed by a Bonferroni post-hoc test. P values of less than 0.05 were taken as being statistically significant.

### 2.5 Computational modelling

Similar to very recent work from our laboratory [36], computational modelling was conducted using the recent cryo-EM open pore structure of hERG [PDB: 5VA1] [26]. A homology model of a hERG closed pore state was also built onto the rat EAG (rEAG) closed pore cryoEM structure [PDB: 5K7L] [37] using Modeller 9.17 [38], with Procheck [39] to assess model quality (see also [36]). The coordinates of the rEAG-based closed state hERG model are available on request. Structural figures were made using PyMOL version 1.4 (Schroedinger, LLC, New York, NY).

## 3 Results

### 3.1 Effects of the I560T mutation on the I_hERG_ current-voltage relation and voltage dependence of activation

The insets to Fig. 1A and B show the voltage protocol used to elicit I_hERG_, which was similar to that applied in prior hERG channel studies from our laboratory (e.g. [15, 28]). From a holding potential of -80 mV, 2s duration depolarising test commands were applied in 10 mV increments to potentials of -40 mV and more positive, followed by a repolarisation step to -40 mV in order to observe I_hERG_ tails [15, 28]. Fig. 1A shows typical records of WT I_hERG_ elicited by this protocol. Pulse currents increased with the magnitude of the test command up to ∼0 mV and declined at positive voltages; accordingly the I-V relation for WT I_hERG_ showed a region of pronounced negative slope at positive test potentials (Fig. 1C). Moreover, resurgent I_hERG_ tails were visible (Fig. 1A). With the same protocol, I560T I_hERG_ during the command pulses increased up to ∼+20 mV, was larger in magnitude than that for WT I_hERG_ and was followed by tail current that was smaller in magnitude than that during the preceding depolarising command pulse (Fig. 1B). Rectification was evident at more positive voltages for I560T I_hERG_ than for the WT channel current (Fig. 1B, C) and comparison of the end-pulse I-V relations for WT and I560T (Fig. 1C) showed the current for the mutant to be significantly greater than that for the WT channel over most of the tested voltage range. Fig. 1D shows normalized current-voltage plots for I_hERG_ tails, which were fitted by Eq. (1) to obtain parameters for voltage dependent activation of the two channels. The derived half maximal activation voltage (V_0.5_) for WT and I560T I_hERG_ were respectively -21.07 ± 1.29 mV (n = 8) and -21.43 ± 1.41 mV (n = 17; p > 0.05), although there was a significant difference in the slope (*k*) of the fitted activation relation (6.61 ± 0.15 mV for WT versus 12.55 ± 2.14 mV for I560T; p < 0.01). In summary, application of a standard I-V protocol to WT and I560T I_hERG_ showed an increase in current density with the I560T mutation, with no significant alteration to voltage dependent activation of the channel.

The presence of overlapping activation and rapid inactivation during depolarisation to positive membrane potentials makes it difficult to quantify the time-dependence of I_hERG_ activation during depolarising commands and, instead, an “envelope of tails” protocol is typically used (e.g. [6, 32]). The inset to Fig. 1E shows the envelope of tails protocol used to compare the rate of I_hERG_ activation for WT and I560T I_hERG_. Tail currents elicited following the different duration voltage commands were normalized to the maximal tail current obtained during the protocol and then the resulting mean data plotted against pulse duration as shown in Fig. 1E and fitted with Eq. (2) (Methods)). The mean time-constant of I_hERG_ activation in WT conditions was 65.4 ± 3.5 ms (n = 5) and that for I560T was 19.6 ± 1.7 ms (n = 7; p < 0.0001). Thus, in the presence of the I560T mutation, I_hERG_ activation was accelerated.

### 3.2 Effects of the I560T mutation on inactivation properties of I_hERG_

The voltage-dependence of inactivation of I_hERG_ was determined in WT and mutant conditions using the protocol shown in Fig. 2 [15, 28]. After an initial depolarising command to +40 mV to activate and inactivate I_hERG_, brief (2 ms) repolarising commands to a range of membrane potentials (in 10 mV increments down to -140 mV) were used to alleviate I_hERG_ inactivation to differing extents and then the amplitude of the rapid I_hERG_ transient during the third step (to +40 mV) was measured. Fig. 2B and C show for WT and I560T conditions expanded portions of the resulting current records, highlighting the rapid I_hERG_ transients elicited by the third step, whilst Fig. 2D show mean normalised plots of I_hERG_ availability (with the amplitudes of currents during the third step normalised to the maximal current obtained, and then plotted against repolarisation potential, as described previously [15, 28]). The data in Fig. 2D were then fitted with Eq. (3), to obtain inactivation V_0.5_ and *k* values. For cells expressing the WT channel, I_hERG_ inactivation was typically half-maximal between -60 and -70 mV (mean V_0.5_ of -66.25 ± 1.91 mV and *k* = 20.15 ± 0.62 mV, n = 6), whilst for I560T hERG, inactivation was positively shifted (mean V_0.5_ of -41.79 ± 3.47 mV and *k* = 25.31 ± 1.22 mV, n = 13; p < 0.001 and p < 0.01 respectively versus WT for both). Thus, voltage dependent inactivation of I_hERG_ was positively shifted by ∼+24.5 mV for I560T I_hERG_. It is also evident from the current traces in Fig. 2B,C that the rate of development of inactivation was slower for I560T than WT I_hERG_ and this was quantified by monoexponential fitting of the decline of I_hERG_ transients following repolarisation steps to -120mV. For WT I_hERG_, the mean inactivation time-constant was 2.31 ± 0.17 ms (n = 6), whereas for I560T I_hERG_ it was 4.72 ± 0.36 ms (n = 13; p < 0.01). In contrast, the time-course of recovery of inactivation (obtained using the protocol shown in the inset of Fig. 2E [34]) did not differ significantly between WT and I560T I_hERG_, with recovery time-constants respectively of 1.84 ± 0.08 ms (n = 10) and 2.05 ± 0.17 ms (n = 18; p > 0.05).

### 3.3 Effects of the I560T mutation on the fully activated I-V relation and the I_hERG_ “window”

**Fig. 3:**
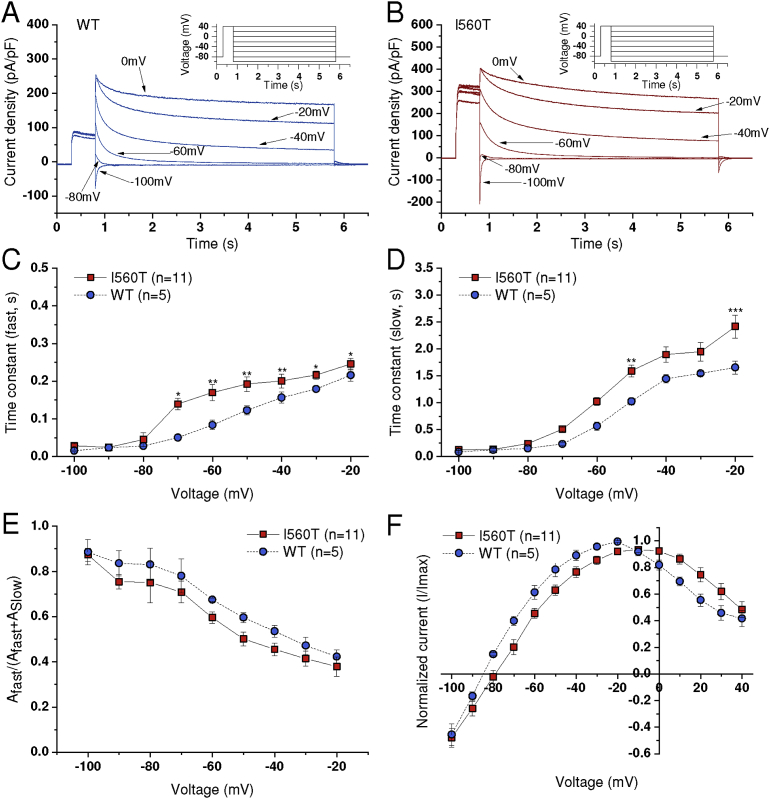
*Fully activated I-V relations and the I*_*hERG*_*‘window’.* (A, B) Representative traces for WT (A; Blue) and I560T (B; Red) elicited by the protocol shown in insets with only selected sweeps shown for clarity. (C, D) Fast (***τ***_f_) and slow (***τ***_s_) time constants of deactivation calculated using Eq. (4). Significant difference from control at p < 0.05, p < 0.01 and p < 0.001 are represented by *, ** and *** respectively. (E) Plots of the fraction (A_f_/A_f_ + A_s_) of fast deactivating current (fitted by the ***τ***_f_) against voltage, from same experiments as C,D. (F) Fully activated I-V relations, calculated with currents normalized to the peak current recorded during the protocol for each cell.

The fully activated I-V relation for I_hERG_ was elicited by the protocol shown in the insets to Fig. 3A and B [15,28]. From a holding potential of -80 mV, the membrane potential was stepped to +40 mV for 500 ms, after which voltage commands were applied for 5 seconds at 10 mV steps between -100 mV and +40 mV. As can be seen in the representative traces of Fig. 3A and B, WT tail current peaks rose as expected [15, 28] as the membrane potential became less negative; these peaked at -20 mV, with I560T I_hERG_ following a similar pattern, albeit with a slightly more positive peak at -10 mV (Fig. 3F). By fitting the tail current decline at each repolarisation voltage with Eq. (4), the time constants for the fast (***τ***_f_) and slow (***τ***_s_) components of deactivation were obtained. As can be seen in Fig. 3C and D, ***τ***_f_ and ***τ***_s_ were modestly increased for I560T I_hERG_ compared to WT hERG at some voltages. This indicates a slight, but significant decrease in the rate of deactivation for the mutant channel. It is notable that neither ***τ***_f_ nor ***τ***_s_ was significantly altered at -80 or -90 mV (i.e. close to the likely resting potential of ventricular myocytes) and so it is unlikely that I_hERG_ deactivation would be significantly slowed by the I560T mutation during diastole. The relative proportions of fast and slow deactivating I_hERG_ were unaltered by the I560T mutation (Fig. 3E). This voltage protocol also revealed a modest but statistically significant positive shift in the reversal potential (E_rev_) of I_hERG_: for WT I_hERG_ E_rev_ was -85.05 ± 0.53 mV (n = 5), whilst that for I560T was -80.34 ± 1.4 mV (n = 11; p < 0.001; Fig. 3F). Using a modified Goldman-Hodgkin-Katz equation, the relative Na^+^:K^+^ permeability ratio was found to change from a WT hERG value of 0.010 to 0.016 for the mutant channel, indicating a modest decrease in the mutant channel's selectivity for K^+^ over Na^+^ ions (cf [15]).

**Fig. 4:**
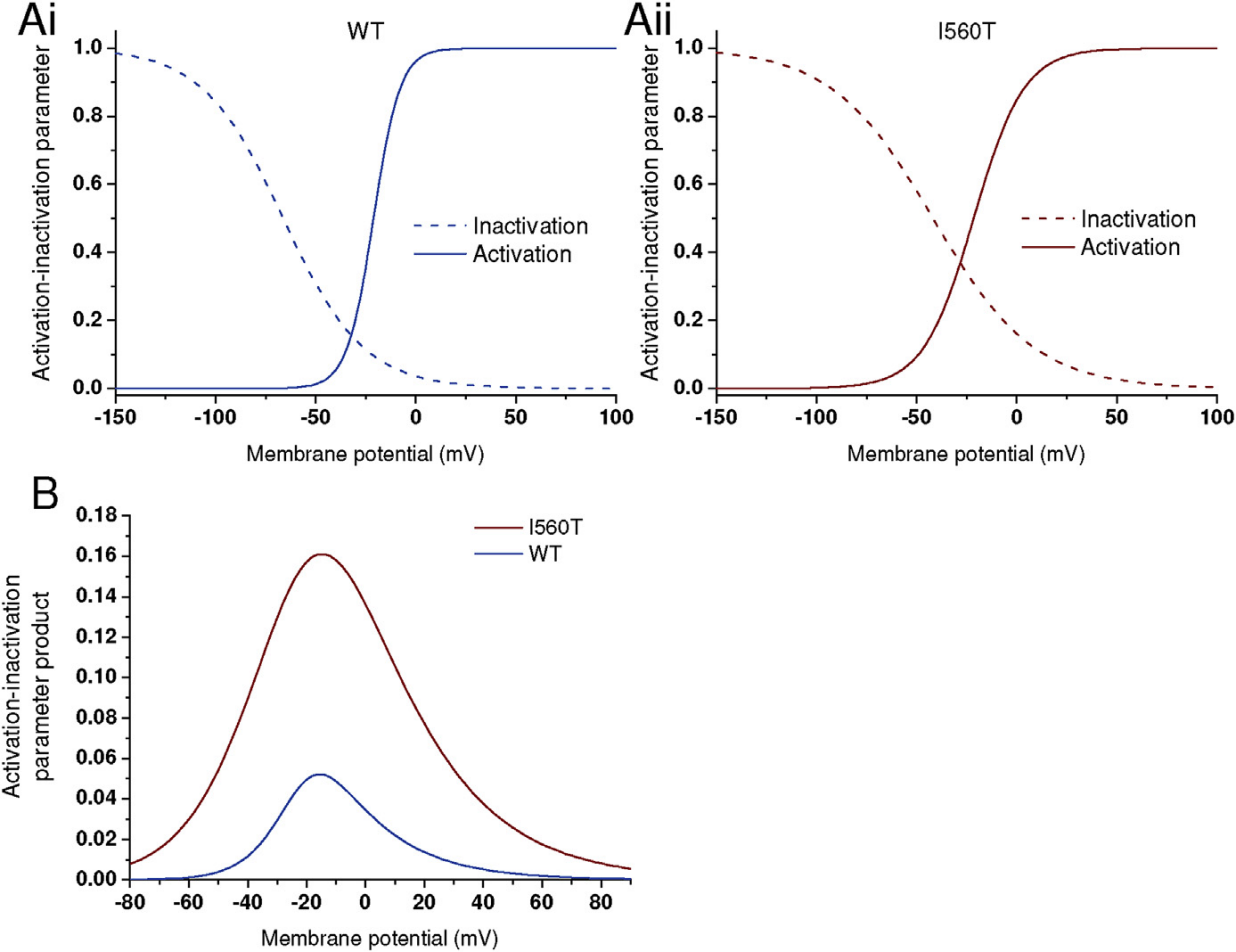
*I*_*hERG*_*window current.* (A, B) Superimposed activation and inactivation (availability) curves for WT (blue) and I560T (red) I_hERG_ respectively. Activation and inactivation parameters were calculated at 2 mV intervals, using the V_0.5_ and *k* values obtained from fitting experimental data. (C) Product of activation and inactivation parameters plotted against voltage to show steady state WT and I560T I_hERG_ window.

Fig. 4 shows the effects of the I560T mutation on the steady-state I_hERG_ window. Window current was calculated as described in the Methods and plots of steady state activation and inactivation parameters were calculated at 2 mV intervals and are shown in Fig. 4A. For WT and I560T I_hERG_, the product of activation and inactivation parameters at each voltage was plotted as shown in Fig. 4B to give the I_hERG_ window current (cf [28]). The I_hERG_ window was substantially larger for I560T I_hERG_ than for WT I_hERG_.

### 3.4 Effects of the I560T mutation under action potential clamp

**Fig. 5:**
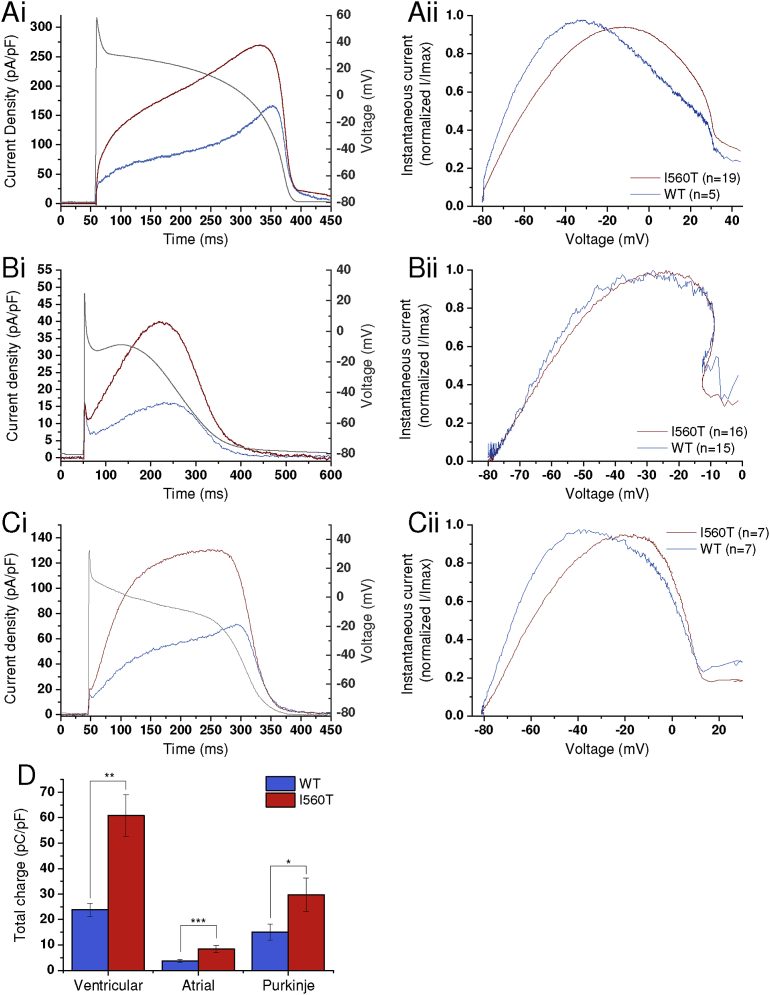
*WT and I560T I*_*hERG*_*response to action potential voltage clamp (AP clamp).* (A) Mean I_hERG_ profile from 19 I560T (red) and 5 WT (blue) AP clamp experiments (Ai) and corresponding instantaneous I-V relations (Aii) during ventricular AP. (B) Mean I_hERG_ profile from 16 I560T (red) and 15 WT (blue) AP clamp experiments (Bi) and corresponding I-V relations (Bii) during atrial AP. (C) Mean I_hERG_ profile from 7 WT (blue) and 7 I560T (red) AP clamp experiments (Ci) and corresponding instantaneous I-V relations (Cii) during Purkinje fibre AP. For all I-V relations, for each cell currents from 5 ms onwards following the initial AP upstroke were normalized to the peak current recorded during the repolarising phase of the AP and mean values plotted. (D) Comparison of the total charge conducted during each action potential, calculated by integrating the current traces, normalized to cell capacitance. Statistically significant differences at p < 0.05 and p < 0.01 are represented by * and ** respectively.

Fig. 5 compares WT and I560T I_hERG_ under action potential (AP) voltage clamp, during ventricular, atrial and Purkinje fibre AP voltage commands. Fig. 5Ai shows overlays of WT and I560T I_hERG_ superimposed on a ventricular AP command (as used previously in [28, 34]), showing augmented current for I560T I_hERG_ during the entire repolarising phase of the AP. Fig. 5Aii shows plots of the normalised instantaneous I-V relations for WT and I560T I_hERG_, showing that maximal I_hERG_ occurred at -29.7 ± 2.7 mV for WT hERG (n = 5) and at -11.9 ± 3.4 mV (n = 19; p < 0.05) for I560T hERG. Fig. 5D compares current integrals for WT and I560T I_hERG_, showing that the total charge carried during the ventricular AP command was ∼2.6 fold greater for I560T than for WT hERG (p < 0.01). Fig. 5B shows similar data during an imposed atrial AP voltage command (as used previously in [27]). Fig. 5Bi shows that, similar to the situation with the ventricular AP command, I_hERG_ was augmented throughout AP repolarisation in the I560T condition, corresponding to a ∼2.3 fold increase in charge in Fig. 5D (p < 0.001). However, the lower AP plateau phase of the atrial AP was associated with less extensive activation of I_hERG_ (evident in the smaller current integrals for the atrial than ventricular AP in Fig. 5D) and the voltage at which maximal I_hERG_ occurred during repolarisation was not significantly different between WT and I560T conditions (Fig. 5Bii; −24.0 ± 2.5 mV (n = 15) and -24.4 ± 2.3 mV (n = 16) for WT and I560T respectively (p > 0.05)). Fig. 5C shows similar data for a Purkinje fibre command (as used in [40]). Again, I_hERG_ was augmented for I560T compared to WT I_hERG_ (Fig. 5Ci) with a ∼2.2 fold increase in charge carried during AP repolarisation (Fig. 5D). Similar to the situation with the ventricular AP command, maximal I_hERG_ occurred earlier during the AP with the I560T mutation, reflected in a more positive voltage at which maximal current occurred (-17.5 ± 3.5 mV for I560T (n = 7) compared to -35.1 ± 0.8 mV for WT (n = 7; p < 0.01)). In summary, the I560T mutation resulted in increased I_hERG_ during all three AP command waveforms, with current also peaking earlier during ventricular, Purkinje fibre, but not atrial AP commands.

### 3.5 Sensitivity of I560T hERG to quinidine

**Fig. 6:**
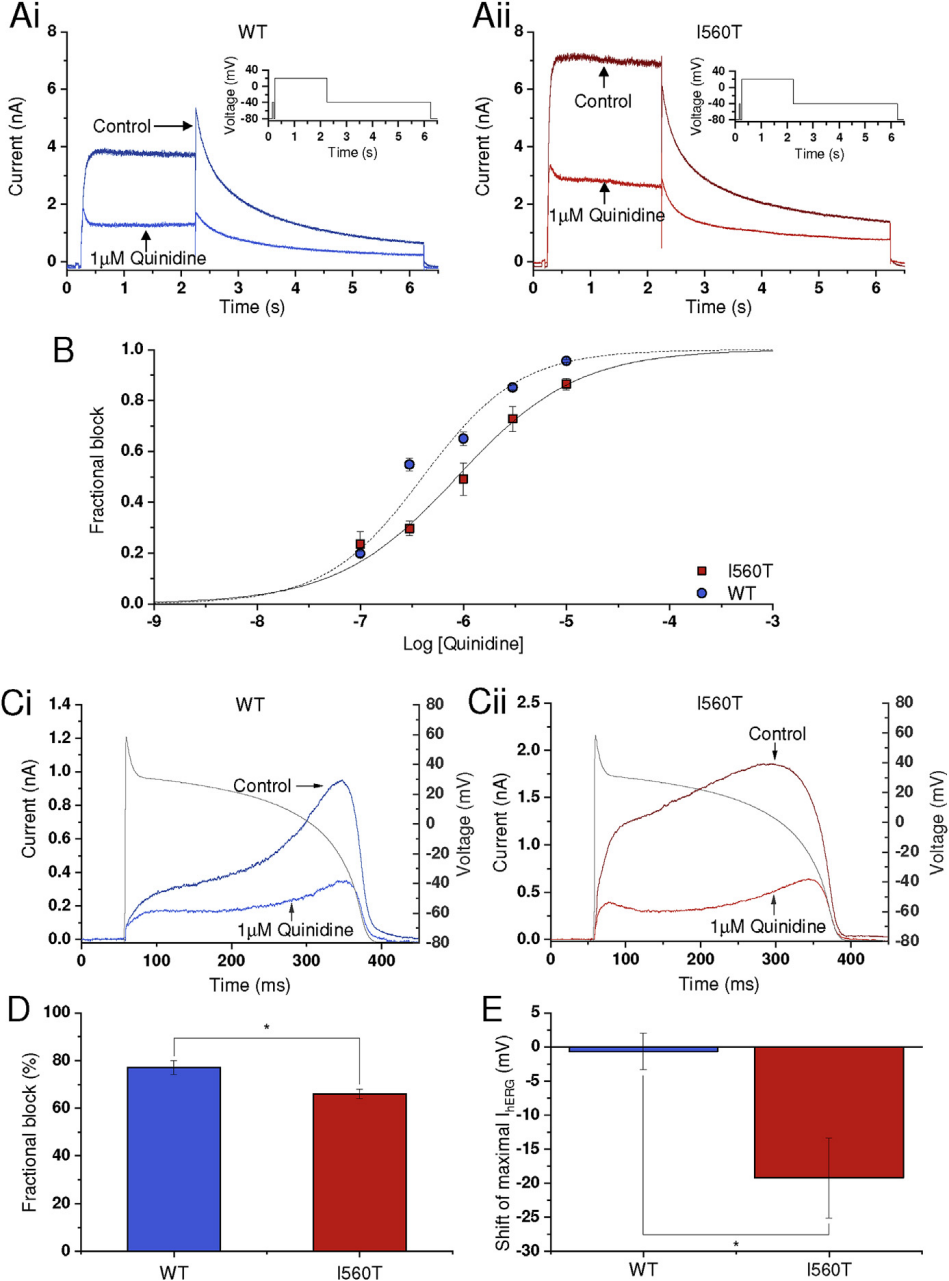
*Response of WT and I560T I*_*hERG*_*to quinidine.* (Ai, Aii) Representative traces of WT (blue) and I560T (red) I_hERG_ respectively, elicited by the protocol shown as the lower traces in each panel and recorded in the absence (black) or presence (grey) of 1 μM quinidine. The interval between successive applications of the protocol was 12 s. (B) Concentration-response relations for inhibition of WT and I560T I_hERG_. Fractional inhibition of I_hERG_ by the range of concentrations shown was calculated using Eq. (5), with a minimum of 5 cells used for each concentration of drug. IC_50_ and Hill coefficient values were calculated using Eq. (6) and are given in the Results. (Ci,Cii) Effects of 1 μM quinidine on I_hERG_ elicited under AP clamp for WT I_hERG_ (Ci; blue) and I560T (Cii; red). AP commands were applied at 1 Hz. (D) Bar chart comparing extent of quinidine inhibition of maximal I_hERG_ during repolarisation (n = 7 for WT and 6 for I560T I_hERG_). (E) Bar chart comparing effect of quinidine on voltage at which peak I_hERG_ occurred during AP repolarisation, expressed as the mV shift in peak current, for WT (n = 7) and I560T (n = 6) I_hERG_. (* denotes statistical significance at p < 0.05).

(Hydro)quinidine has been used successfully in the treatment of congenital SQTS [11, 41, 42, 43], with relatively modest effects on its hERG blocking potency reported for the N588K and T618I hERG mutations [16, 17, 19, 20]. In order to establish quinidine's effectiveness against I560T I_hERG_ the voltage protocol shown in the insets to Fig. 6Ai and Aii was used. This protocol has been employed in multiple prior studies of I_hERG_ pharmacology from our laboratory (e.g. [17, 28, 29]). Fig. 6Ai, Aii show the effects of 1 μM quinidine on WT and I560T I_hERG_ respectively. In both conditions, quinidine produced substantial I_hERG_ inhibition. Fractional inhibition of I_hERG_ ‘tails’ at -40 mV was calculated by measuring tail current magnitude in control and drug relative to the brief (50 ms) prepulse to -40 mV (as in [17, 28, 29]) and applying Eq. (5). Mean fractional block data at five concentrations were then plotted as shown in Fig. 6B and fitted with Eq. (6) to obtain half-maximal inhibition (IC_50_) and Hill coefficient (*h*) values. For WT I_hERG_, the derived IC_50_ was 0.38 ± 0.03 μM with an *h* value of 0.86 ± 0.06. For I560T, an IC_50_ value of 0.88 ± 0.14 μM (2.3-fold the WT value; p < 0.01) with an *h* value of 0.70 ± 0.09. The I_hERG_ blocking potency of some drugs is sensitive to the stimulus protocol used to activate I_hERG_ [44, 45]. Therefore, additional experiments were performed in which 1 μM quinidine was applied under ventricular AP clamp. As shown in Fig. 6Ci and Cii, the amplitude of I_hERG_ elicited by the ventricular AP command was substantially reduced by quinidine for both WT and I560T I_hERG_. The bar charts in Fig. 6D show mean (±SEM) inhibition of peak I_hERG_ during the ventricular AP command; this was only modestly reduced for I560T compared to WT I_hERG_. We also quantified the reduction in net charge carried by I_hERG_ throughout the AP command (cf [46]), which 1 μM quinidine reduced by 78.5 ± 4.8% (n = 7) for WT hERG and 68.6 ± 3.0% (n = 6) for I560T hERG (p > 0.05 vs WT). Thus, in both conventional voltage clamp and AP clamp experiments, I560T hERG largely retained sensitivity to quinidine. In addition, under AP clamp the voltage at which peak I_hERG_ occurred during AP repolarisation was shifted to more negative potentials for I560T I_hERG_, whilst that for WT I_hERG_ was unaffected (Fig. 6E).

### 3.6 Location of I560 in the hERG channel structure

**Fig. 7:**
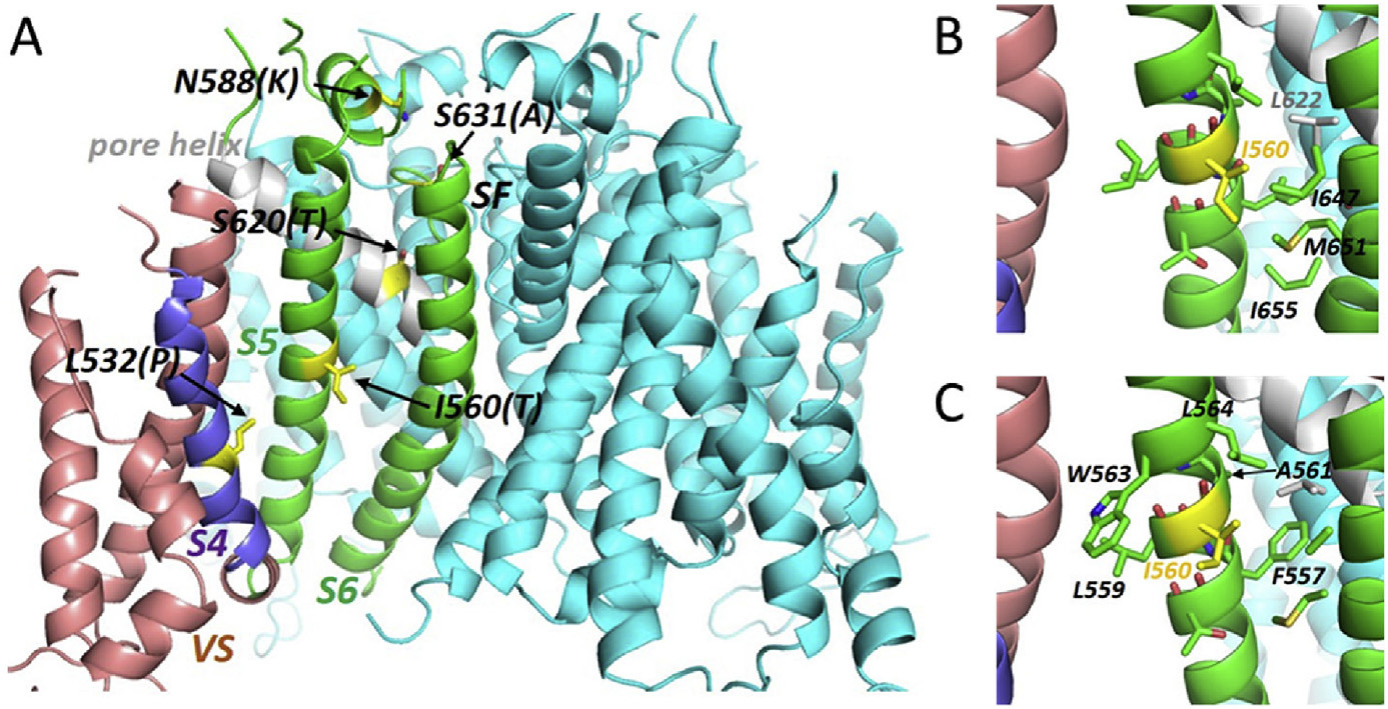
*Structural context of the I560 residue.* (A) Membrane domain of the recent hERG construct cryo-EM structure [26] highlighting I560 and other selected inactivation-attenuating residues. VS: voltage sensor; SF: selectivity filter. The three subunits that are not highlighted/annotated are coloured pale blue. (B) Close up of the S5 helix containing I560 with all atoms within 7 Å of any I560 side chain atom shown as sticks coloured according to their origin (e.g. the side chain of L622 on the pore helix is within 7 Å and is coloured grey). I560 makes no interactions with residues on the voltage sensor domain including S4. (C) A similar conclusion arises from inspection of a hERG homology model built on the cryo-EM structure of EAG [37]. Amino acid annotations are residues on S5 near I560 that do potentially interact (C-C distances within 5 Å) with residues on S1 (L559; W563), the pore helix (A561; L564) and S6 (F557).

**Fig. 8:**
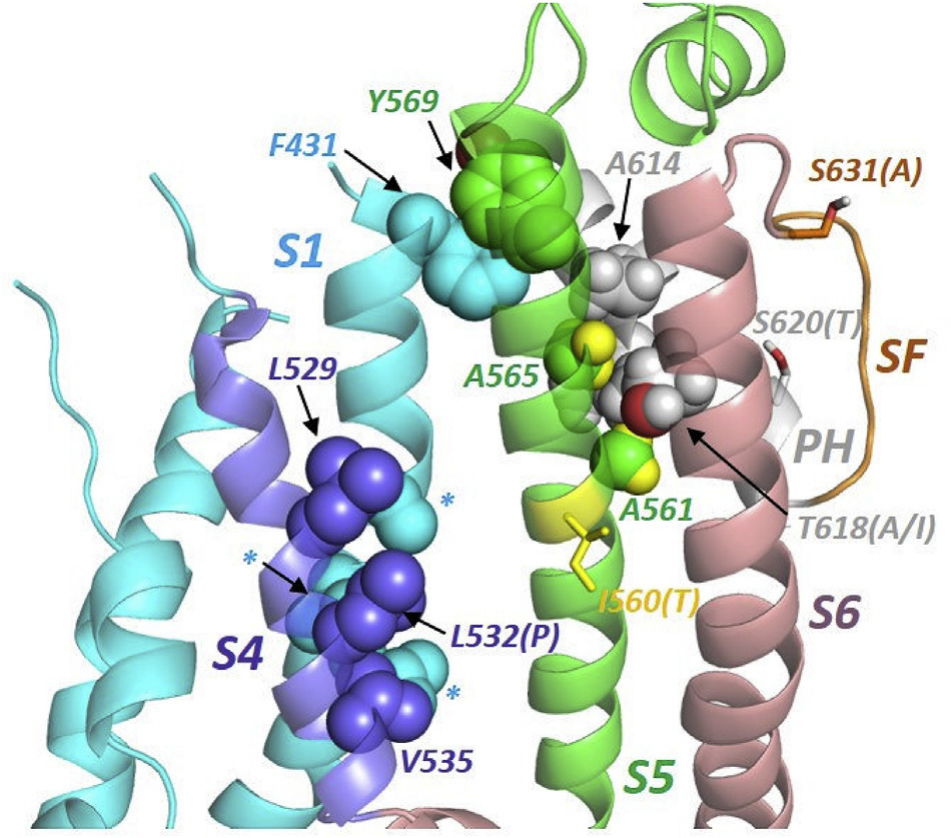
*Helix-helix interactions potentially relevant to hERG gating.* Hydrophobic side chains on the voltage sensor S4 helix interact closely with sides chains on S1 (A422, V418, L415 indicated by asterisks for clarity) but not with S5. S1 interacts with the pore domain *via* a cluster of aromatic amino acids involving S1 (F431), S5 (Y569) and the pore helix (PH; Y611 hidden from view). S5 interacts with the pore helix via side chain “knobs into holes” packing involving A561 and A565 on S5 (green spheres with yellow hydrogens) and A614, L615, T618 and F619 side chains on the pore helix. The S5-pore helix interface is immediately adjacent to the selectivity filter (SF). hERG mutations that perturb inactivation gating are indicated with mutation in parentheses. Hydrogens have been omitted from side chains highlighted in the voltage sensor for clarity.

Our experimental data indicate that the I560T mutation produces multiple effects on hERG channel kinetics: accelerating activation, slowing inactivation and positively shifting the voltage dependence of inactivation, whilst also modifying ion selectivity of the channel. Prior work has suggested hydrophobic, energetic coupling between S5 and S4 helices of hERG during inactivation involving residue I560 [24]. A structure of the open hERG channel has recently been reported using cryo-electron microscopy (cryo-EM) [26], whilst a closed-pore state of the closely related EAG channel has also been captured [37]. We have recently constructed a closed channel model of hERG based on the related rEAG structure [36, 37]. Fig. 7 shows the location of the I560 residue in open and closed hERG channel conformations. Fig. 7A shows I560 on the S5 helix in the context of the full membrane domain along with the location of other residues that attenuate hERG inactivation. Expanded regions around the centre of the S5 helix in the hERG structure and the rEAG-based hERG model are shown in Fig. 7B and C, respectively, along with atoms that fall within 7 Å of any of the I560 side chain atoms. In both the hERG structure and rEAG-based model I560 is oriented towards the membrane bilayer and makes no direct interactions with residues in other protein domains apart from an interaction with the long side chain of M651 on S6 (Fig. 7B,C; this is the only remaining close approach if the distance constraint is reduced from 7Å to 5Å). We were not able to substantiate prior interpretations from homology modelling based on the structure of Kv1.2/2.1 chimeric channels [24] that I560 is proximate to hydrophobic residues on the S4 domain of an adjacent subunit in the tetramer. If present, such interactions would have been identified by light blue atom stick representations in Fig. 7B and C. The absence of interdomain S5-S4 interactions is a consequence of the non-domain-swapped arrangement of subunits in the hERG (and EAG) structure, but the new cryoEM structures also demonstrate that direct *intra*subunit interactions between I560 on S5 and S4 residues do not occur at least in the membrane-depolarized states captured in the cryoEM structures. The most intimate interactions with the S5 helix involve close packing between S5 and the pore helix where the side chains methyl groups of A561 (adjacent to I560) and A565 slot between the side chains on the pore helix (Fig. 8), which is noteworthy given the role of pore helix residues in I_hERG_ inactivation and the proximity of this region to the selectivity filter (Fig. 8). In summary, consideration of the I560T mutant in the context of available information on hERG channel structure indicates that I560 appears to face membrane lipid and to make no direct interaction with nearby structural elements including the S4 helix, but has the potential to influence interactions between S5 and the pore helix.

## 4 Discussion

### 4.1 Effects of the I560T mutation on I_hERG_ in context

The results of this study indicate that the effects of the I560T mutation on I_hERG_ are more extensive than those reported when this mutation was first implicated in the SQTS [12]. Our results accord well with those of Harrell and colleagues in respect of increased I_hERG_ magnitude and lack of alteration to half-maximal activation voltage of I_hERG_ with the mutation. Harrell and colleagues did not comment on any effect of I560T on the slope factor for I_hERG_ activation, however, whilst we observed a modest though statistically significant difference between WT and I560T *k* values, with more variability in this parameter for the mutant than WT channel (as shown by the respective SEM values). The positive shift in voltage-dependent inactivation seen in the present study (+24 mV) is larger than that reported in the initial report of the mutation (+14 mV) [12] and is comparable to those produced by the L532P (+32 mV) [28] and T618I (+25 mV) [20] mutations that have been investigated under similar conditions to the present study. It is notable, however, that the WT inactivation V_0.5_ value for I_hERG_ from the study by Harrell and colleagues was relatively positive (-27.3 mV) in comparison with that in the present and other studies [14, 15, 28, 47], which might have led to an underestimation of the differences between WT and I560T I_hERG_ in [12]. The reasons for the differences between the two studies in this regard are not known: both were conducted at physiological temperature, but differences in expression system (HEK 293 versus COS-7) and voltage protocol may have contributed. Alterations to other kinetics parameters (activation/deactivation rates, inactivation rate) and ion selectivity seen here for the I560T mutation have not previously reported [12] and demonstrate an influence of the I560T substitution on multiple properties of the hERG channel.

The I560 residue in ERG is well-conserved across species [12] and has been previously linked to inactivation gating of hERG via putative hydrophobic interactions with the voltage sensor S4 helix [24] (dark blue in Figs. 7 and 8). Leucine and valine residues on the S4 helix (L529, L530, L532 and V535) have been proposed to be key molecular determinants of inactivation gating [24]. Rate equilibrium free energy relationship (REFER) analysis has led to the proposition that conformational rearrangements of the S5 helix and S5-Pore linker precede that for S4, which in turn occurs before that for S6. Double mutant cycle analysis combining each of L529S, L530S, L532S and V535S with the I560A mutation showed their effects to be non-additive, leading to the conclusion of energetic coupling between S4 and S5 residues [24]. In a hERG homology model based on the Kv1.2/2.1 crystal structure, hydrophobic residues on S4 and S5 of adjacent subunits faced one another, leading to a conclusion that energetic coupling is likely to occur via hydrophobic interactions [24]. A surprising conclusion from the present study is that if the I560 residue is examined in the context of the cryoEM hERG structure [26], it does not face an adjacent S4 domain in the gated state; this is also the case for a closed channel model built on rEAG (Fig. 7). In fact the S5 helix does not make any interactions with S4 in the cryoEM structures of hERG and EAG (Figs. 7 and 8).

An alternative explanation for the gating effects of the I560T mutation is threonine-induced bending of the S5 helix [48]: the polar hydroxyl side chains of threonine and serine interact poorly with lipid chains and may preferentially hydrogen-bond with the backbone carbonyl oxygen of amino acids three or four residues towards the N-terminus in helices (T556 and F557 in the case of I560T in hERG) as commonly found in transmembrane helices of membrane proteins (e.g. [48, 49, 50]). This interaction induces a small bend in the helix that would be expected to perturb S5 interactions in I560T hERG. The most intimate interactions with the S5 helix involve close packing between S5 and the pore helix where the side chains methyl groups of A561 (adjacent to I560) and A565 slot between side chains on the pore helix (Fig. 8). Minor disruption of this interface by mutation at I560 is likely to influence channel gating and potentially ion selectivity since the pore helix plays a fundamental role in the dynamics of hERG inactivation [51].

In fact, inspection of the hERG cryoEM structure suggests a common theme in the transmission of effects of inactivation-perturbing mutations in the S4, S5 and pore helices to the selectivity filter. Rather than direct interactions with S5 as previously suggested [24], the S4 helix residues L529, L532 and V535 interact directly with residues on the voltage sensor S1 helix, at least in the voltage sensor activated state (Fig. 8). Effects of mutation on S4 are therefore expected to be transmitted to the selectivity filter via the S1 helix most likely through a cluster of aromatic amino acids (F431 on S1, Y569 on S5 and Y611 on the pore helix) that interact closely at the extracellular ends of the S1, S5 and pore helices (Fig. 8). We previously showed that the inactivation-perturbing mutation L532P [28] perturbs the conformation of the S4 helix [52] and this should affect the S4-S1 helix interactions. Thus each of the inactivation-perturbing mutations L532P, I560T, T618A/I lie within a network of helix interactions involving S4-S1, S1-S5 and S5-PH (Fig. 8) and transmission of gating effects from the voltage sensor to the selectivity filter is likely to involve these interactions rather than direct S4-S5 helix interactions. Previously, the N588K SQT1 mutation in the S5-Pore linker (which is spatially distinct from the pore) has been seen not only to disrupt inactivation gating, but also to produce modest change in pNa:pK ratio [15]. By analogy, effects of changes to S5 on interactions with the pore helix, or mediated via the S5-Pore linker, could influence ion selectivity.

### 4.2 Clinical relevance of the findings of this study

The patient in whom the I560T mutation was initially identified presented with atrial fibrillation and flutter, and his short QT_c_ interval became evident following catheter ablation treatment for supraventricular arrhythmia. Thus the presenting arrhythmias were *supraventricular*, although the patient's father and brother had undergone sudden death [12]. Simulations performed to probe the consequences of the changes to I_Kr_ due to the I560T mutation reproduced abbreviation of *ventricular* repolarisation and showed increased susceptibility to ventricular fibrillation, which is consistent with sudden death reported in the patient's family. Simulations were not included to investigate effects of the mutation on atrial electrophysiology [12]. The findings of our study indicate that more extensive alterations to *in silico* representations of I_hERG_/I_Kr_ are likely to be required to recapitulate accurately *in silico* the mutant's effects at the channel level and consequently upon cell/tissue simulations. Moreover, our AP clamp data both provide clear evidence that the I560T mutation can accelerate repolarisation of ventricular and supraventricular APs and provide a resource for accurate validation of *in silico* of the mutation's effects (cf [53]). Both magnitude and timing of I_Kr_ during ventricular and Purkinje fibre APs are likely to be altered by the I560T mutation, whereas during atrial APs, the principal effect is anticipated to be on current magnitude. The more than doubling of I_Kr_ magnitude is highly likely to abbreviate both the atrial AP and effective refractory period (ERP), thus facilitating re-entrant atrial arrhythmia, which is consistent with the AF and flutter seen in the index patient. Increased current magnitude and earlier timing of peak repolarising current is anticipated to abbreviate AP duration and ERP in both ventricular and Purkinje fibre cells. Previously, the increase in I_hERG_ caused by the N588K hERG SQT1 mutant was found to be greater during ventricular than Purkinje APs [14, 34], which may contribute towards heterogeneity of repolarisation and ventricular arrhythmia substrate. Here, the increase in net charge during ventricular and Purkinje fibre APs was 2.6 and 2.2 fold respectively. A comparative simulation approach is required to establish whether or not the extent of the difference between the two translates into increased heterogeneity between Purkinje fibre and ventricular AP repolarisation in the I560T setting. Moreover, through the systematic incorporation in, and omission from, simulations of the different kinetic effects and altered ion selectivity seen here for I560T hERG, it should be possible to determine which are most significant for the SQTS phenotype with this mutation.

To our knowledge, drug sensitivity of the I560T hERG mutation has not been probed previously. Long term follow-up of SQTS patients found quinidine to be more effective in patients with the SQT1 variant than in non-SQT1 patients [41]. The N588K mutation, which profoundly alters I_hERG_ inactivation, increases the IC_50_ for quinidine inhibition of I_hERG_ block by 3.5–5.8 fold [16, 17] and the drug retains effectiveness in patients with that mutation [41, 54]. Recent simulation data provide evidence that the beneficial effects of quinidine on ventricular repolarisation in N588K-linked SQT1 are attributable to its actions on hERG, whilst effects to prolong refractoriness also involve its Na channel actions [55]. Here, the IC_50_ for I560T I_hERG_ block by quinidine was 2.3 fold that of WT I_hERG_ and, under AP clamp, the ability of 1 μM quinidine to reduce I_hERG_ and the current integral during the AP was little affected by the I560T mutation. In addition, quinidine mitigated the positive shift in voltage dependence of peak I_hERG_ during the AP command. For the T618I hERG SQT1 mutation quinidine has previously been suggested to shift voltage-dependent inactivation and the “fully-activated” I-V relation towards those of WT I_hERG_ [19]. Similar effects could potentially account for the negative shift in peak I560T I_hERG_ during AP repolarisation seen here with quinidine. Consistent with this notion, the instantaneous voltage dependence of WT I_hERG_/I_Kr_ during ventricular AP repolarisation is known closely to match the I_hERG_ fully-activated I-V relation [56] and the voltage dependence of I560T I_hERG_ during the ventricular AP in the presence of quinidine became closer to that of the WT channel (Fig. 6C). Plasma levels of quinidine can readily reach micromolar levels during daily dosing regimens [57] and considering our results in this context we would anticipate that quinidine is likely to retain its ability to reduce I_Kr_ amplitude in I560T-linked SQT1. The fact that Class Ia drugs are effective in many (though not all) cases of SQT1 [9,11,41,43,58] is likely to reflect the fact that they are much less dependent on hERG channel inactivation to bind than are high affinity drugs such as methanesulphonanilides [18, 59, 60]. The smaller attenuation of quinidine's action by the I560T than by the N588K mutation (which shifts inactivation by +60 to +90 mV [14, 15]) is also consistent with this. The patient in whom the I560T mutation was found refused an implantable defibrillator and it is not stated as to whether or not he received antiarrhythmic drug treatment. However, quinidine is likely to be beneficial in reducing the increased I_hERG_/I_Kr_ and, potentially, also in restoring the current's timing during the ventricular AP in this form of the SQTS.

### 4.3 Limitations

The present study used homozygous expression of I560T hERG, to enable direct comparison with the characteristics of I_hERG_ with this mutation first reported by Harrell *et al*, who used homozygous mutant expression conditions [12]. However, the proband in whom the I560T hERG mutation was identified was heterozygous for the mutation [12]. Whilst there is ample precedent for *in vitro* investigations of heterozygous hERG mutations in SQTS using homozygous expression conditions [8, 14, 15, 16, 17, 18, 19, 20, 46], heterozygous subunit composition may lead to quantitative difference in the effects of the mutation to those reported here and previously [12] using homozygous expression. For example, the use of concatemeric channels has shown graded effects of the S631A mutation on I_hERG_ inactivation [61]: tetrameric channels incorporating a single S631A subunit showed a positive inactivation V_0.5_ shift that was 69% of that for the homozygous tetramer, whilst incorporation of a second S631A subunit only modestly increased this to 73% of that for the homozygous channel [61]. Future work is warranted to compare consequences of heterozygous and homozygous expression of the I560T mutation. In respect of the effect of the I560T mutation on quinidine block of I_hERG_, heterozygous expression might be expected to result in an inhibitory potency that lies between those for WT and homozygous expression conditions (for which in any case the alteration here was relatively small). Notably, predictions of retained effectiveness of quinidine and disopyramide based on homozygous expression have correlated well with patient observations for the N588K mutation, which produces a much larger voltage shift in inactivaction kinetics to that seen here for I560T [8, 16, 17]. So, the fact that the I560T mutation had only a small effect on quinidine potency is significant.

In common with most investigations, this study relied on recordings from hERG1a channels. Increasing evidence suggests that native I_Kr_ may be comprised of both hERG1a and the shorter hERG1b isoform [62, 63]. To our knowledge, only one previous study has systematically investigated the effects of any SQT1 mutation using hERG1b; it found that the attenuation of I_hERG_ inactivation by the N588K mutation was amplified for hERG1a/1b heteromeric channels [64]. Whether or not this could also occur for the I560T mutation merits future investigation. Similarly, whether changes seen for hERG alone (here and previously [12]) may be influenced by co-expression with potential accessory subunits (KCNE1/KCNE2) also remains to be determined.

## 5 Conclusions

The I560T mutation increases I_hERG_ magnitude and positively shifts voltage dependence of I_hERG_ inactivation, whilst slowing the rate of development of inactivation and accelerating activation time-course. The gain of function produced by the mutant is anticipated to increase I_Kr_ during atrial, ventricular and Purkinje fibre APs, accelerating repolarisation and thereby abbreviating refractoriness. Whilst our data do not preclude conformational coupling between I560 and S4 residues, the channel structure seems inconsistent with direct hydrophobic interactions between the two. The cryoEM structures of hERG and rEAG indicated that any such conformational coupling is likely to be indirect and mediated by interactions amongst S1, S5, and the pore helix. The reduction in potency of quinidine block of I_hERG_ by the I560T mutation is sufficiently small that the drug is likely to be of value in limiting the increase in magnitude and altered timing of I_Kr_ in SQT1 caused by this mutation.

## Declarations

### Author contribution statement

Andrew Butler: Performed the experiments; Analyzed and interpreted the data; Wrote the paper.

Yihong Zhang, Christopher E. Dempsey: Conceived and designed the experiments; Performed the experiments; Analyzed and interpreted the data; Wrote the paper.

Jules C. Hancox, A. Graham Stuart: Conceived and designed the experiments; Analyzed and interpreted the data; Wrote the paper.

### Funding statement

This work was supported by Sudden Arrhythmic Death Syndrome UK (SADS UK; AGS and JCH) and the British Heart Foundation (JCH and CED; PG/15/106/31915 and PG/17/89/33414).

### Competing interest statement

The authors declare no conflict of interest.

### Additional information

No additional information is available for this paper.
